# Shapes of You? Investigating the Acceptance of Video-Based AAL Technologies Applying Different Visualization Modes

**DOI:** 10.3390/s23031143

**Published:** 2023-01-19

**Authors:** Julia Offermann, Wiktoria Wilkowska, Caterina Maidhof, Martina Ziefle

**Affiliations:** Human-Computer Interaction Center, Chair of Communication Science, RWTH Aachen University, 52074 Aachen, Germany

**Keywords:** technology acceptance, video-based AAL technology, visualization modes, quantitative study, human factors

## Abstract

An aged population, increasing care needs, and a lack of (in)formal caregivers represent major challenges for our society today. Addressing these challenges fuels efforts and developments in innovative technologies leading to various existing AAL applications aiming at improving autonomy, independence, and security in older age. Here, the usage of video-based AAL technologies is promising as detailed information can be obtained and analyzed. Simultaneously, this type of technology is strongly connected with privacy concerns due to fears of unauthorized data access or inappropriate use of recorded data potentially resulting in rejection and non-use of the applications. As privacy-preserving visualizations of video data can diminish those concerns, this empirical study examines the acceptance and privacy perceptions of video-based AAL technology applying different visualization modes for privacy preservation (n = 161). These visualization modes differed in their degrees of visibility and identifiability, covering different levels of privacy preservation (low level: “Blurred” mode; medium level: “Pixel” and “Grey” modes; high level: “Avatar” mode) and are specifically evaluated based on realistic video sequences. The results of our study indicate a rather low acceptance of video-based AAL technology in general. From the diverse visualization modes, the “Avatar” mode is most preferred as it is perceived as best suitable to protect and preserve the users’ privacy. Beyond that, distinct clusters of future users were identified differing in their technology evaluation as well as in individual characteristics (i.e., privacy perception, technology commitment). The findings support the understanding of potential users’ needs for a successful future design, development, and implementation of video-based, but still privacy-preserving AAL technology.

## 1. Introduction

Tackling the rising challenges related to demographic change, efforts have been made to develop innovative approaches, such as Ambient Assisted Living (AAL) technologies, aiming at support and assistance of older people and people in need of care [1,2,3]. In addition to sensor- and audio-based technologies, video-based devices such as thermal cameras, RGB cameras, or RGB-D devices have been more frequently used in recent years. These applications provide the options to monitor an environment continuously reporting detailed visual information [4]. In this regard, progressions in computer vision and underlying algorithms have enabled the development of smart cameras in terms of intelligent vision-based systems: these systems are applied in diverse areas but being are promising in health care and health monitoring [4,5,6,7]. Those approaches are not only able to stream video in real-time, but can be used to extract useful information from the visual data and provide that information to specific groups of people. In the field of AAL, such video-based technologies can be used for diverse health applications, e.g., analyses of human behavior and respective changes, recognition of activities of daily living, physiological monitoring, rehabilitation, gait analysis, promotion of healthy lifestyles, especially also for the detection and prevention of falls and emergencies [4,8,9]. Enabling such a broad spectrum of functions, video-based AAL technologies have a large potential to support people in their everyday life and to increase autonomy, independence, and safety in older age.

Beyond the potential and the advantages the usage of AAL technologies might bring, barriers and concerns, in particular regarding an intrusion of privacy and data security, exist [10,11,12]. In particular, the application of cameras can be seen as intrusive to the individual user’s privacy due to concerns in terms of unauthorized access or inappropriate use of video data. Previous research in this regard has already shown that cameras and video-based technologies are perceived and considered to be more critical compared to other technical approaches [13,14].

Technical developments and adaptions offer the option to address privacy concerns by applying diverse visualization modes that conceal visual details of the recorded footage [15]. Thus far, previous research [16] analyzed participants’ perceptions of such visualization options based on pictures of the modes in a rather abstract and general manner. However, a realistic analysis and understanding of users’ attitudes, intentions, and decisions to use or not use critically perceived technical applications, such as video-based AAL technology at home, is essential: only in this way can a user-centered development and design can be realized, and concrete recommendations for a successful implementation of such devices in older age be derived. Therefore, this study aims at a realistic evaluation of the perceptions and acceptance of specific visualization modes of video-based AAL technologies based on their real video sequences. Thereby, specific preferences for different situations of daily living, acceptance, affective evaluations, and the influence of user diversity are investigated.

### 1.1. Ambient-Assisted Living Applications and Video-Based Systems

With regard to the technical developments, there are rather fluent transitions between the areas of assisting systems, lifelogging approaches, assistive smart home technologies, and Ambient Assisted Living: these approaches share that they are conceptualized, developed, and designed for support in older age in terms of activity and behavior monitoring, detection of emergencies, (medical) reminding of purposes, and relief in everyday life (for diverse overviews, see [1,3,4]). In addition to health-improving functions, such approaches can be used to improve health, to enable a longer independent life at an older adult’s own home, and to provide support for different stakeholders in older age (i.e., older and frail people themselves but also their professional or family caregivers) by collecting and analyzing health-related data material [1,3,17]. Thereby, there is a huge variety of technologies and systems, reaching from small wearable sensors to ambient installed systems integrating different types of sensors that are being applied in private homes as well as in professional care contexts (e.g., hospitals, nursing homes, rehabilitation facilities). These approaches can be used to fulfill a broad spectrum of functions, e.g., recognition of social activity [18], improving health in terms of motivation for being physically active [19], recognition and analyses of single activities and movements for health monitoring [20], and identification of changes in movements or behaviors (e.g., as symptoms for dementia) [21]. Beyond that, one key function of numerous AAL approaches aims to detect emergencies, such as falls and dangerous situations, in order to enable early notifications for doctors and caring persons or even prevent such situations [22,23]. An approach that has been followed increasingly for several years focuses on the integration of video-based systems within the home environment of older adults aiming at support and assistance [4,17]. Thereby, current video-based applications not only allow recording situations in real-time but enable the extraction of useful information from the video data based on intelligent systems and underlying algorithms [5,6]. This way, detailed analyses of gaits or human behaviors, as well as the detection of emergencies, falls, and other dangerous situations, have just been made possible [4] considering very diverse user groups.

To sum up, a variety of applications are aimed at the support and assistance of people in their private home environments based on intelligent monitoring and analyses by means of computer vision. Thereby, the technology is considered to be used to generate information about the users, i.e., especially older people in need of care, allowing risk detection and support services and simultaneously protecting the users’ privacy as best as possible. Addressing the latter, Padilla-López et al. [15] proposed visual privacy with a context approach, addressing the trade-off between preserving privacy, on the one hand, and the intelligibility of the recorded material, on the other hand. Within their approach, privacy protection is achieved by applying different visualization modes, i.e., types of disclosure of the respective information concealing sensitive information and providing different levels of protection depending on the individual context. Thereby, the individual context is composed in each case by the observer (having authorized access to the video material, the identity of the monitored person, the relationship between the monitored person and the observer (e.g., relatives, doctors, care personnel), the monitored person’s appearance, the location of monitoring (e.g., bathroom), and the monitored activities or detected events (e.g., eating, undressing, falling). The authors [15] proposed eight different visualization modes enabling diverse levels to preserve privacy: raw image, blur, pixelating, emboss (grey), silhouette, skeleton, 3D avatar, and invisibility. The modes provide different levels of privacy preservation by illustrating the visual characteristics of the monitored person differently with regard to the pose and shape of the monitored person and/or related to the colors and textures. The idea behind the concept is that the monitored user can select the most preferred visualization mode depending on the context, i.e., in particular, related to certain situations or activities, or depending on the observer. The user is able to grant diverse permissions for different observers and to select different visualizations for certain contexts. Hence, a trade-off is enabled between abandoning some of the individual privacy in favor of increasing security if the respective situation or context is important enough. In addition to the technical potential of this approach, the permission of future users, and thus their acceptance and willingness to use it, are necessary.

### 1.2. Perception and Acceptance of Video-Based AAL Technologies

Reaching from wearable sensors in clothing, smart technologies to be taken wherever desired, up to ambient systems integrated into private environments and institutional care facilities, assisting technologies can now accompany us almost everywhere. But are we also willing to accept them? Do we want them around us everywhere and at all times? In which situations are they helpful, and when are they a nuisance? These questions are crucial when it comes to the use of technologies in different contexts.

In the process of technology adoption, technology acceptance is an undenied key player. Research in this area that originates not only from the area of information systems but also from psychology and sociology has resulted in several theoretical models: the most established technology acceptance models are the Technology Acceptance Model [24] and the Unified Theory of Acceptance and Use of Technology [25], as well as their extensions. Over the years and parallel to the technology development, these models have served as a framework for a vast number of studies focusing on the understanding of how users develop their acceptance for new technologies to facilitate their adoption. In addition, research has shown that the users’ characteristics (e.g., age, gender, experience) play a decisive role in the adoption process, making it evident that technology acceptance can also differ among different user groups (=user diversity). As of today, despite the great amount of litrature on the subject, there is not a one-fits-all-model that would be universally applicable, but the acceptance of information processing devices, applications, or systems is highly context-dependent and thus has to be examined separately.

In addition to these generally acknowledged acceptance models and criteria, former research has also identified that context-related perceptions of benefits and concerns regarding a particular technology considerably shape its acceptance and long-term adoption [10,26]. Jaschinski (2014) [27] identified that, especially for older individuals and persons in need of care, among the most relevant motives for using assisting technologies are an autonomous life, social involvement, health and safety, and support with the activities of daily living. In the context of aging in place, individuals frequently acknowledge health- and safety-related applications detecting emergencies or falls, enabling quick access to health data, and giving notifications in case of emergencies (e.g., [26,28]). Reminder functions for medication, rehabilitation, or appointments (e.g., [29]), as well as the overall reduced burden for a caring family, were further benefits enhancing the general acceptance and intended use of assistive technologies in older age [10,30].

On the other hand, concerns associated with assistive technologies can significantly reduce the willingness to use them. The most important empirically elaborated barriers having a high contradicting impact on technology acceptance are the uncertainty regarding data security, stigmatization, lack of human interaction, loneliness, and costs (e.g., [16,27]). One of the biggest barriers, however, seems to be the concern of one’s own privacy—we illustrate this currently highly discussed topic in more detail hereafter. Other studies in this research area revealed that perceived surveillance, loss of control, or unauthorized access to sensible (health) data by third parties represent further obstacles to an accepted technology use [31,32].

Thus, the acceptance issue when using assistive technologies in living environments is a decisive one, especially for technology generations that barely spent a (large) part of their lives dealing with digitization and data processing [33]. In a previous study [16], we also found that decisions about the use, and therefore acceptance, of video-based applications are significantly influenced by deeply anchored cultural and societal phenomena and can differ across cultures. This research further corroborated—in line with previous studies in this area (e.g., [32,34])—a reluctant willingness to log video-based data in the own home environment and a generally weakly developed intention to use cameras, independently of the modes of information presentation (different visualization alternatives).

Due to its enormous role in acceptance, the sensitive issue of privacy in the use of video-based data is addressed in more detail below.

### 1.3. Privacy Perceptions as a Necessity of Privacy Preservation

Definitions of privacy are either value-based and described as a human right or economic commodity or cognate-based, referring to privacy as a behavior or a predisposition of the individual to behave [35]. As part of this behavioral approach, privacy is considered a state of mind [36,37] or an assertion of control [37,38,39]. As part of these cognate-based definitions, various authors distinguish between several dimensions or types of privacy to account for the multi-dimensionality of the construct (e.g., see [37,40,41]). As such, Westin [37] distinguishes between solitude (i.e., being completely isolated), intimacy (i.e., reducing sensory input from outsiders to focus on personal relationships), anonymity (i.e., freedom from recognition and personal surveillance) and reserve (limiting self-disclosure). Burgoon [41] depicts privacy in four dimensions, namely informational, social, psychological, and physical. According to the author, whether privacy is high or low depends on the ability to control the specific aspects of privacy within these dimensions. In line with this, Altman [42] conceptualizes privacy as a boundary regulation process through which individuals always try to achieve an optimum level of social interaction in each moment. Thereby, privacy regulation depends on both personal factors (e.g., individual need for privacy, interpersonal skills, personality, and the ability to utilize privacy control mechanisms) as well as situational factors (e.g., physical environment including location, barriers, presence and willingness of other individuals) [43]. In short, privacy is contextual [44,45]. These regulatory processes of privacy management explain the mechanisms of “how” privacy is reached.

Furthermore, privacy plays a crucial role when it comes to personal well-being, positive human functioning and effective management of social interactions [42,43,46,47]. To that end, privacy regulation has been identified to serve several purposes, such as contemplation, autonomy, rejuvenation, confiding, and creativity. It is these identified needs that correspond to the reasons “why” people seek privacy [46,48]. Naturally, these privacy needs cannot be met if privacy regulation management fails. As a consequence, such a mismatch between the desired level of privacy and the actual amount of privacy evokes concerns and may cause either loneliness or a severe invasion of privacy, potentially leading to depression or anxiety [42,49]. Especially within digital environments, the boundary regulation process is more challenging, easily leading to perceptions of loss of control over private personal information [48]. Therefore, when linking digital environments to privacy, individuals focus more on protection against privacy menaces than the fulfillment of their actual privacy needs [48]. Technology enabling pervasive surveillance, storage of a huge amount of data and a fast-paced and global distribution of information is indeed a major threat to privacy [45].

Similarly, in the field of video-based AAL, privacy is mainly regarded as a concern comprising feelings of permanent surveillance, fear of access and misuse of personal information, and obtrusiveness, which all possibly hinder the adoption of such intelligent (visual) systems [10,11,50,51,52]. However, privacy is multifaceted in nature and highly dependent on context, including situational and personal factors, even when it comes to video-based AAL monitoring. Caine et al. [53] report that the comfortableness of being monitored with a camera varies depending on the activity being performed. Particularly, activities that have sensitive or personal notions, such as sexual activity, hygiene care, or taking a shower, were considered as being most uncomfortable to be recorded through visual health monitoring [53]. The performed activity is most often bound to the situation. Situational factors, such as environmental constraints, play a crucial role in privacy perception, and findings on video-based AAL acceptance suggest [54] that the acceptance of medical monitoring with cameras declines the more private the monitored spaces are. Interestingly, older and ill participants accepted visual systems more than healthy participants, having fewer fears of losing one’s dignity and privacy [13]. Indeed, the need for care [52] and the degree of disability [55], as well as perceptions of safety and security [56,57], may contribute positively to the acceptance of AAL technology. Overall, these findings show that privacy is malleable and—given specific situational and personal circumstances—may be regulated through a trade-off with several benefits that come with visual monitoring, such as increased safety and helpfulness [52,56,57]. Most often, it is the technological features that contribute to this trade-off process diminishing privacy concerns. For instance, participants of a qualitative study with programmable video monitoring for fall detection at night [58] particularly appreciated that certain time slots could be chosen for monitoring and that video material was processed automatically and images were blurred. These features impacted privacy perception positively and preserved the perception of control over technology. In fact, technological efforts in computer vision to mitigate (older) adults’ concerns and ideally fulfill privacy needs even during pervasive monitoring of more intimate activities and during elevated care are increasingly being made and reached with de-identification [59] privacy-by-design [60] and privacy-by-context [15] frameworks (for reviews, see [4,61]). In the context of video-based AAL, computer vision efforts focus on both the preservation of the privacy of body images, the so-called bodily privacy [62,63] and (to a lesser extent) on identity protection through visual privacy preservation methods, such as among other visual obfuscation [61]. On the user level, it was examined [16] how potential users of video-based lifelogging technology evaluated these different visualization modes of the camera output. Results revealed that the real image output was rated as the most privacy intruding, whereas visualization modes, i.e., the silhouette and the skeleton image, were rated as least privacy invading. On the other hand, among five different options, the real image was considered the most optimal representation. However, none of the visualization modes was rated as being a particularly good optimal representation, and generally, participants showed a rather reluctant behavioral intention to use these visualizations.

In general, the importance of privacy and its preservation within the context of video-based AAL cannot be denied and many technological efforts are being developed to adhere to user preferences and perceptions.

### 1.4. Research Gaps, Aim, and Questions

In addition to the technical potential of privacy-preserving approaches, such as the development of visualizations allowing different levels of visibility and identifiability [15], the permission of future users, and thus their acceptance and willingness to use these approaches, are necessary and have to be examined. Beyond the first research in this regard [16], the investigations of acceptance have to be deepened by critically examining the perceptions of different visualization modes in everyday situations and in addition to the general acceptance in different user groups (e.g., younger vs. older people, males vs. females, healthy vs. chronically diseased people, etc.). Here, it would be worth investigating which user factors characterize different evaluation patterns of using video-based AAL technology. Further, empirical research, with not only images but realistic video sequences and examinations of opinions on these filter options, is necessary to gain a better understanding of privacy details and their impact on technology acceptance.

To address these research gaps, it was the aim of this empirical study to reach a specific and realistic evaluation of video-based AAL technologies applying different visualization modes addressing adults of all ages. For this purpose, different visualization modes were evaluated based on real video sequences the participants watched prior to the evaluations of the single visualization modes. The underlying research questions were the following:

1. RQ1: How are video-based AAL technologies perceived and accepted in general?
2. RQ2: Which specific visualization modes are selected for different exemplary situations of everyday life (in older age)?
3. RQ3: Are there differences in the acceptance of the different visualization modes?
4. RQ4: Are the visualization modes and their characteristics evaluated differently?
5. RQ5: Are there user groups differing in the acceptance of video-based AAL technology? How are the groups and their evaluation patterns characterized?

## 2. Materials and Methods

In the following section, the empirical approach of the conducted study is presented, introducing the design and the concept of the online survey. Further, we describe the characteristics of the sample and the procedures of data analysis.

### 2.1. Empirical Design

The idea of this study was to enable the evaluation of video-based AAL technologies in general as well as specifically in terms of the assessment of different visualization modes being applied for video-based AAL technologies. To realize these evaluations, an online survey was conceptualized, aiming at reaching a broad sample of adults covering all ages. The survey’s structure with its constructs and their items are described in Section 2.2.

For the general evaluation of video-based AAL technologies, a descriptive scenario was prepared for the participants explaining the use and functions of a video-based AAL system. It was aimed at providing technically correct information in a simple, comprehensible, and transparent manner. The scenario served as a basis to introduce the participants to video-based AAL technologies enabling the evaluation of their perspectives and opinions on the general idea of video-based AAL technologies. The applied descriptive scenario was conceptualized as follows:

“*The system described here aims at everyday support in older age and works with the help of cameras. The cameras are installed in the residents’ own homes and can record their daily lives. The cameras are about the size of a hand and are mounted at head height and in various rooms. They record only the image but no sound, so they cannot listen to conversations. The system and its cameras can be switched off and on by the user at any time. The cameras, or the system behind them, can offer users various health services. These include, for example, the detection of falls, support during rehabilitation (e.g., after an operation), remote monitoring by doctors or nurses, or the early detection of signs of dementia or frailty. The cameras continuously evaluate the video material. In this way, the system can independently detect an accident or emergency and then trigger an emergency call. With the user’s consent, it is also possible to record important events to learn why they happened - for example, what caused a fall. The more videos that are recorded, the more likely it is that the system will be able to detect, for example, whether a person is suddenly performing unusual movements or actions. If this indicates a risk, e.g., in the case of persons with incipient dementia, family members or the family doctor can be notified.*”

**Figure 1:**
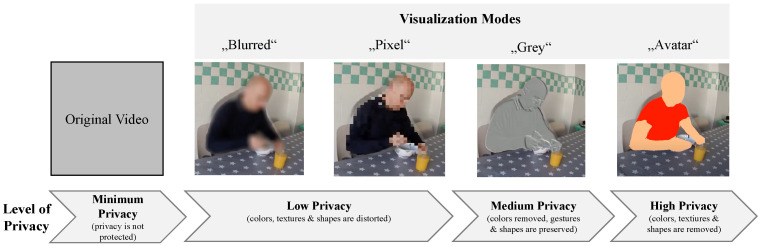
Overview of the visualization modes and their privacy level based on and adapted from [15]: video sequences recorded and provided for the study by F. Florez-Revuelta and P. Climent-Pérez (University of Alicante).

In the second step, the assessment of different visualization modes being applied for video-based AAL technologies was focused on. As described before, previous research has investigated the perception of different visualization modes based on pictures [16]. To enable a realistic experience and evaluation, we examined in this study the acceptance of a selection of visualization modes based on video sequences compared to the original video. We chose four visualization modes addressing different levels of privacy preservation: a low level (here: “Blurred”), a medium level (here: “Pixel” and “Grey”), and a high level (here: “Avatar”) of privacy protection in comparison to the original video, in which the users’ privacy is not at all protected (see Figure 1).

Before the participants evaluated the visualization modes, a scenario was given to them in order to introduce the visualization modes and to enable the participants to better empathize with the situation of having a video-based AAL system in their own home environment. For this purpose, the following explanations and scenarios were used:

“Using a lifelogging system in your own home comes with several advantages and disadvantages. For example, installing cameras may raise privacy concerns. Such concerns may arise because your family members, doctors, or caregivers may be watching you in situations where you do not want them to. To mitigate these privacy concerns, various video visualization modes have been developed to make people unrecognizable on the video. The following is a brief explanation of the filters and their characteristics. Please read this explanation carefully.

*Imagine that you have decided to use a video-based AAL system for support in older age at home. The system now provides you with different visualization modes, each offering a different level of privacy and visibility. Before installing the cameras, you can select a mode that will make you unrecognizable on the video footage. Once installed, the cameras now record what’s happening in your home around the clock. The moment someone wants to access your videos, the visualization mode you selected will be activated. The access can be from one of your relatives, your doctor or nurse, for example, because they want to check your status or because the system has sent a warning - like in case of a fall. The visualization mode you choose now processes the recorded video so that your caregivers can only see the video in the way you want them to. In doing so, the visualization modes are designed to protect your privacy while allowing your family members, doctors, or caregivers to assess whether something has happened to you. The stronger a visualization mode is, the more detail is obscured from the image. While with some visualization modes you can still make out colors, structures and shapes, other modes only show the person’s posture or figure. The more details are rendered unrecognizable, the less information can be extracted from the video by relatives, doctors, or nurses.*

The next step is to evaluate the visualization modes. For this purpose, short video sequences are shown below, which present the filters in different situations. As mentioned at the beginning, there are no wrong answers, you are asked for your personal feeling with regard to the videos and their visualization modes. We will start with a concrete application scenario of the lifelogging system and its cameras. Please watch the following video. Imagine that the camera is installed in your home and that you are the person being filmed here.”

After the participants read the scenario and the instructions, they started to watch the videos for three situations of daily living: “Falling” as a concrete use-case and frequent situation in older age, “Eating” as a typical activity of daily living, and “Undressing” as an example for a sensitive situation in the everyday life. For each situation, first, the original video was shown. Afterward, four videos were presented, applying the different visualization modes in a randomized order.

The evaluation of the visualization modes followed after the presentation of all videos for the three situations. Here, the participants evaluated their perception of the three situations and selected their most preferred visualization mode for each situation. Related to each visualization type, the participants evaluated their perception as well as their acceptance of the visualization modes in terms of an intention to use them.

The applied scenarios, as well as the structure and items of the online survey described in the next section, have been pre-tested by three persons of different ages. These participants confirmed the comprehensibility, in particular, with regard to the scenarios and reported an average response time of 25 min.

### 2.2. Online Survey

**Figure 2:**
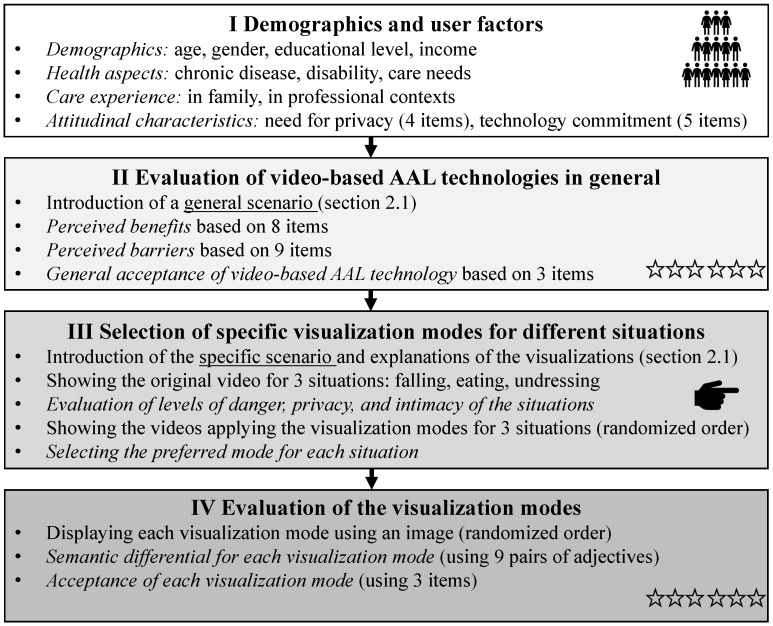
Overview of the key components of the online survey (see Section 2.1).

The online survey was set up in the German language and was structured into four parts: (i) demographics and user factors, (ii) evaluation of video-based AAL technologies in general, (iii) selection of specific visualization modes for different situations, and (iv) evaluation of the visualization modes. To support the understanding of the process, the key components of the online survey are illustrated in Figure 2.

**(i) Demographics and user factors**. Following the introduction that provided an overview of the study’s aim and intention, the first part focused on demographic data and user factors. In addition to demographic data, such as age, gender and education level, various personal attitudes were collected. Among others, these were needed for privacy (measured with 4 items; α=0.60, e.g., “I feel uncomfortable when strangers have insight into my home”) and technology commitment (measured with 5 items based on [64]; α=0.86, e.g., “I am very curious about new technical developments”). Subsequently, the participants answered questions regarding health- and care-related aspects. In more detail, they indicated if they suffer from a chronic disease and if a physical disability or restriction is presented (answers: yes/no, optional indication of which diseases or restrictions). In addition, it was asked if help in everyday life is needed (answer options from “always” to “never”). Further, the participants indicated if they have previous experiences in care, either in the family, the social environment or in a professional context (answer options: yes/no).

**(ii) Evaluation of video-based AAL technologies in general**. This part of the survey started with a descriptive scenario explaining the use and function of a video-based AAL system (see Section 2.1). Following the scenario, the participants evaluated different potential benefits associated with the usage of the system (8 items; α=0.91, e.g., “…it enables me to live independently in my home”). In addition to the perceived benefits, perceived barriers were evaluated using 9 items (α=0.82, e.g., “…I would feel monitored by the system”). All single items of the perceived benefits and barriers are illustrated in the results (Section 3.1). The general acceptance of a video-based AAL system was operationalized as the intention to use the system based on three items (α=0.76, e.g., “I would use a video-based AAL system for support in my home”). As for the personal attitudes, the benefits, barriers, and acceptance were assessed on a six-point Likert scale (min = 1: “I totally disagree”; max = 6: “I totally agree”).

**(iii) Selection of specific visualization modes for different situations**. In this part of the survey, the participants were first introduced to the privacy-preserving video-based technology; here, the different visualization modes were presented. Within a short introduction, it was described that privacy and surveillance concerns are well recognized and that video filters have been developed to diminish those concerns. In this regard, a scenario was provided to the participants putting them in the role of a potential user of the video-based AAL system (see Section 2.1). Within the scenario, a choice of different visualization modes was provided to the participants to make them unrecognizable on the video footage. Here, it was explained to the respondents that the visualization modes allow different degrees of visibility. The user is able to predefine the desired visualization modes so that the respective visualizations are started automatically at the moment when somebody accesses the video. Thereby, it was emphasized that the more details are made unrecognizable, the less information can be taken from the video and can thus be beneficially used. Having the scenario in mind, the participants should evaluate the different visualization modes. In the first step, the four visualizations were evaluated in three different situations (see Section 2.1): “Falling”, “Eating”, and “Undressing”. For each situation, the original video of the situation was shown first, illustrating the situation itself. As a baseline for the perception of these three situations, the participants assessed the levels of danger, intimacy, and privacy on six-point Likert scales (min = 1: low to max = 6: high). Subsequently, the videos were shown again, applying the four visualization modes in a randomized order for each situation. After watching all video options, the participants were asked to select their preferred visualization mode for each of the three situations. Next to the four visualization modes, the participants could also choose the options of not wanting the situation to be filmed at all or wanting the original video without any visualization modes (see Section 3.2). These two options were provided in order to avoid the forced-choice format and to gain more insights into the selection patterns regarding the situations.

**(iv) Evaluation of the visualization modes**. The fourth part of the study aimed at the assessment of the single visualization modes independently. Therefore, each visualization mode was displayed again using an image as a reminder and evaluated by means of a semantic differential and a concrete intention to use the visualization modes. To avoid a valuation bias, the order of the visualization modes was again randomized. Starting with the semantic differential, nine pairs of adjectives were evaluated by the participants on six-point scales (e.g., acceptable (=1) vs. not acceptable (=6)). The semantic differential included aspects referring to the visualization modes’ acceptance, their potential for privacy preservation, technical characteristics, and appeal. All pairs of adjectives are illustrated in Section 3.2. In addition to the semantic differential, the respondents also evaluated their intention to use the single visualization modes. Here, three items were applied in accordance with the overall acceptance of visual AAL technologies for all four visualization modes: “Pixel” (3 items, α=0.78), “Blurred” (3 items, α=0.78), “Grey” (3 items, α=0.77), and “Avatar” (3 items, α=0.76).

At the end of the online survey, thanks were expressed for sharing opinions and participating in the study, and an open comment field was provided for feedback, questions, and suggestions.

For data acquisition, the link to the online survey was shared in social networks, i.e., groups whose topics indicated an interest in the subject of this study, such as interests of nursing, care institutions and the care of family members (e.g., Facebook groups, online forums for seniors, seniors’ websites, and neighborhood forums). In addition, a poster with a QR code leading to the online survey with a brief explanation of the study’s topic and purpose was hung up in places where many people spend their time, such as bakeries, cafés, and the waiting rooms of physicians’ office.

### 2.3. Sample Description

Overall, 260 respondents took part in the study that was conducted in Germany in July 2020, and data collection was open for 24 days. After data cleaning (i.e., focusing on completeness, speed, and quality control), n = 161 data sets remained for further analyses (“N” refers to the whole population of the sample). In the following, “n”, is used to describe subgroups within our sample. the mean age of the participants was 46.80 (SD = 18.74, median = 49), covering an age range between 18 and 86 years. While individuals aged 50 years and older were surveyed for the reason that they are the target users of the technology, younger people served on the one side as potential future users and on the other side as a control group to identify differences in their perception of the object of investigation. The sample consisted of 62.1% female (n = 99) and 37.9% male (n = 61) participants (one participant indicated a diverse gender). The educational level of the participants was high with 50.9% (n = 82) of the participants holding a university degree, 21.7% (n = 35) a university entrance qualification, and 5.0% a doctoral degree. Only 22.4% (n = 36) of the participants reported lower education degrees, i.e., diverse secondary school certificates. Considering health-related aspects, the sample was overall in a good state of health, as only 27.3% (n = 44) of the participants indicated suffering from a chronic disease, and only 13.7% (n = 22) indicated having a physical disability or restriction. Referring to the measured individual attitudes (min = 1; max = 6), the participants generally showed a moderately positive evaluation of their technology commitment (M = 4.08; SD = 1.00) and a confirmation of their privacy perception (M = 4.35; SD = 0.95)—indicating existing needs for protecting and preserving their own privacy.

### 2.4. Data Analysis

All measured items referring to the evaluation of video-based AAL technologies were assessed on six-point Likert scales (min = 1; max = 6), whereas the value of 3.5 represented the mid-point of the scale. Hence, values < 3.5 indicated rejection, while values > 3.5 indicated acceptance of an item. Reliability analyses (Cronbach’s α>0.7) ensured the quality of all measured constructs (here: perceived benefits, perceived barriers, acceptance, acceptance of the specific visualizations modes). In addition to descriptive statistics (means (M), standard deviations (SD), and relative frequencies (%)), repeated measure ANOVAs were used to investigate differences between the four visualization modes (“Avatar”, “Blurred”, “Grey”, and “Pixel”) regarding their acceptance and affective evaluations. Thereby, the F-ratio is reported as a calculated test statistic. Beyond that, a two-step cluster analysis was conducted to identify and investigate distinct evaluation patterns of video-based AAL technologies. Thereby, influences of user diversity on the identified clusters were analyzed using single-factor ANOVAs. The level of significance was set at 0.05, and values above the significance level (*p* > 0.05) were interpreted as not significant (n.s.).

## 3. Results

Within this section, the results of the present empirical study are described, starting with the overall acceptance and perception of video-based AAL technologies. Following that, the evaluations of different visualization modes of video-based AAL technology are presented considering affective assessments as well as selections depending on specific situations of daily living. Finally, it is shown to what extent the evaluations are impacted by user diversity, i.e., individual characteristics of the participants.

### 3.1. Acceptance of Video-Based AAL Technology (RQ1)

The participants assessed the overall Acceptance of video-based AAL technology slightly negatively (M = 3.17; SD = 1.15) based on the mean of the scale (M = 3.5). Operationalized as a behavioral intention to use, the participants tended to slightly reject the two positive items, whereas they simultaneously confirmed the negative item: *I do not intend to use a video-based AAL system for support in my home* (M = 4.26; SD = 1.50).

**Figure 3:**
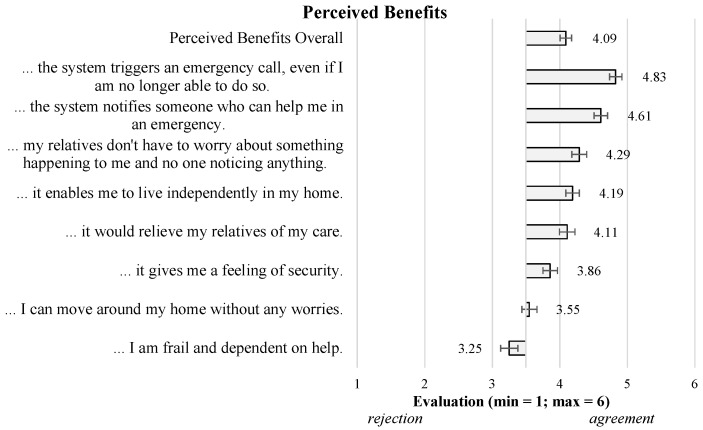
Perceived benefits of using video-based AAL technology (n = 161).

**Figure 4:**
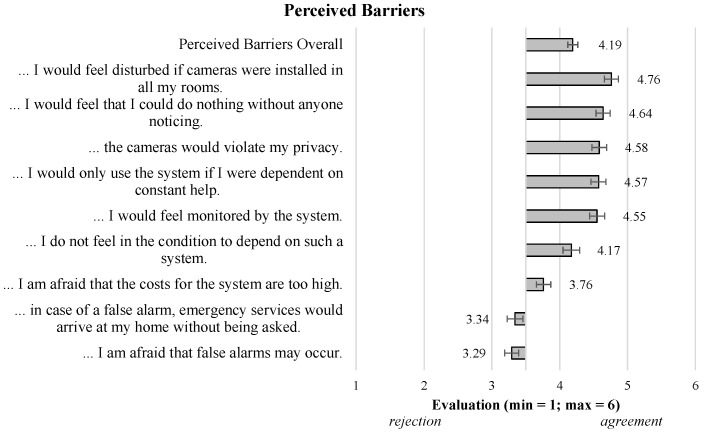
Perceived barriers to using video-based AAL technology (n = 161).

Beyond the acceptance construct, the participants also evaluated the perception of video-based AAL technologies in terms of Perceived Benefits (Figure 3) and Perceived Barriers (Figure 4). Starting with the Perceived Benefits, the results showed overall agreement of the participants (M = 4.09; SD = 1.09), indicating that most of the single aspects were confirmed to represent the benefits of using video-based AAL technology. Thereby, emergency-related benefits received the highest agreement (*…triggers an emergency call…* (M = 4.83; SD = 1.17) and *…notifies someone who can help me…* (M = 4.61; SD = 1.27)). Other aspects, e.g., referring to living *independently in my home* (M = 4.19; SD = 1.26) and *…relieve my relatives of care* (M = 4.11; SD = 1.43), received agreement as well. In contrast, the item *I can move around my home without any worries* (M = 3.55; SD = 1.44) was assessed almost neutrally, while the participants rejected liking to use the system in case they are *frail and dependent on help* (M = 3.25; SD = 1.61).

Moving to the Perceived Barriers, overall, the results showed confirmations of the participants (M = 4.19; SD = 0.92), indicating that almost all single aspects represent “real” barriers of using video-based AAL technology. Four aspects referring to an invasion of privacy received the highest agreements and thus represent severe barriers: *…feel disturbed if cameras were installed…* (M = 4.76; SD = 1.34), *…I could do nothing without anyone noticing* (M = 4.64; SD = 1.32), *…would violate my privacy* (M = 4.58; SD = 1.39), and *…feel monitored by the system* (M = 4.55; SD = 1.40). Further, the participants confirmed that they would only use the system if they *were dependent on care* (M = 4.57; SD = 1.39). Related to this, the participants further agreed with the statement that they *do not feel in the condition to depend on such a system*, indicating that necessity and dependency are relevant usage conditions. Further, the concern that the *costs for the system are too high* (M = 3.76; SD = 1.34) was only slightly confirmed. Finally, concerns related to false alarms (e.g., *…I am afraid that false alarms may occur* (M = 3.29; SD = 1.33) were slightly rejected, indicating that these aspects do not represent severe barriers of using video-based AAL technology.

### 3.2. Comparing Different Visualization Modes

Beyond an assessment of video-based AAL technologies in general, this study aimed at an investigation of different privacy-preserving visualization modes of video-based AAL technology, which were presented to the participants per video examples. The respective results are presented in the following sections.

#### 3.2.1. Selecting Visualization Modes for Different Situations (RQ2)

**Figure 5:**
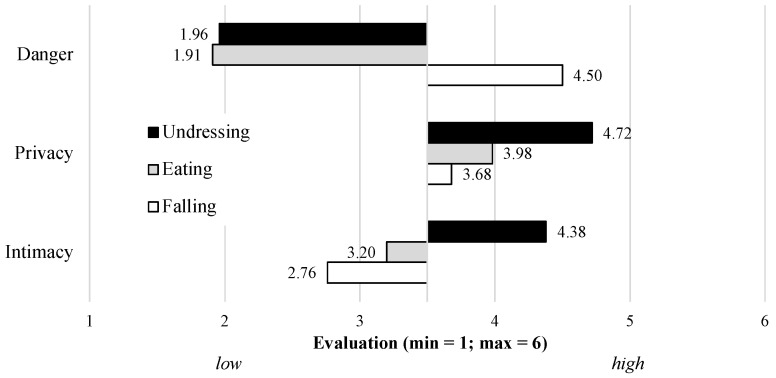
Perceptions of using video-based AAL technology in different situations (n = 161).

To enable a realistic evaluation of the visualization modes as possible, we asked the participants to select the preferred visualization mode within different concrete situations of daily living. As examples, three completely different situations were pre-selected: *Eating* as a typical activity of daily living, *Falling* as an example of a critical situation in older age, and *Undressing* as a presumably very private and intimate activity (see Section 2.1). In the first step, the participants evaluated the degree (min = 1: low; max = 6: high) of privacy, intimacy, and danger depending on the three situations. The results are presented in Figure 5.

Starting with *Eating*, this activity was perceived to be clearly not dangerous (M = 1.91; SD = 1.00), moderately private (M = 3.98; SD = 1.13), and rather not intimate (M = 3.20; SD = 1.28). In comparison, *Undressing* was also not perceived to be dangerous (M = 1.96; SD = 0.89), but to be clearly private (M = 4.72; SD = 0.93) and intimate (M = 4.38; SD = 1.27). Not surprisingly, *Falling* was perceived to be dangerous (M = 4.50; SD = 1.09), private on a rather neutral level (M = 3.68; SD = 1.22), and not to be intimate (M = 2.76; SD = 1.37). These distinct evaluation patterns confirm the three activities to cover a range of differently perceived activities of daily living. Therefore, it is useful to investigate which visualization modes the participants would select and prefer in these situations.

**Figure 6:**
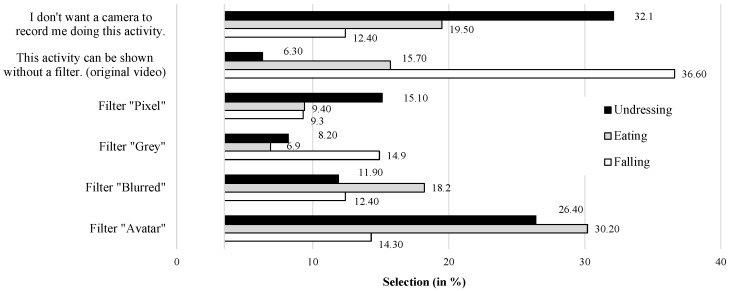
Selection of visualization modes in different situations (n = 161).

Figure 6 presents the selection patterns of the participants depending on the three introduced situations of daily living. Starting with the situation of *Eating*, the majority of the participants selected the “Avatar” visualization mode (30.2%; n = 48), followed by the “Blurred” visualization (18.0%; n = 29). Compared to that, the “Pixel” (9.3%; n = 15) and “Grey” (6.8%; n = 11) modes were chosen by only a few participants. Beyond that, 19.5% (n = 31) selected the option that they do not want a camera to record this activity, while 15.7% (n = 25) selected the opposite opinion, namely that this activity can be recorded and shown without a privacy-preserving visualization (original video).

Moving to the situation of *Undressing*, the selection patterns changed and most of the participants (32.1%; n = 51) chose the option that they did not want a camera to record this activity. Only 6.3% (n = 10) selected the opinion that this activity can be shown by the original video (i.e., without using a visualization mode). Related to the specific visualization modes, the majority of the participants selected the “Avatar” (26.4%; n = 42) mode again, followed by the “Pixel” (15.1%; n = 24) and the “Blurred” (11.9%; n = 19) visualization modes. In line with the evaluations of the activity *Eating*, the “Grey” mode (8.2%; n = 13) was selected the least frequently.

Considering the situation of *Falling*, the selection patterns changed fundamentally. Most of the participants selected that this activity can be shown without a privacy-preserving visualization, indicating that the original video would be accepted (36.6%, n = 59). In comparison, a clearly lower proportion of the participants (12.4%; n = 20) chose the option that they did not want a camera to record this situation. Related to the specific visualization modes, the “Grey” (14.9%; n = 24) and the “Avatar” (14.3%, n = 23) modes were preferred, followed by the “Blurred” (12.4%; n = 20) and the “Pixel” (9.3%; n = 15) visualization modes.

The specific selection of the visualization modes has shown that their evaluation and acceptance depend on the respective context. In the following, it is investigated to what extent the evaluation and acceptance of privacy-preserving visualizations of video-based AAL technologies also depend on the individual characteristics of the potential users.

#### 3.2.2. Acceptance of Different Visualization Modes (RQ3)

**Figure 7:**
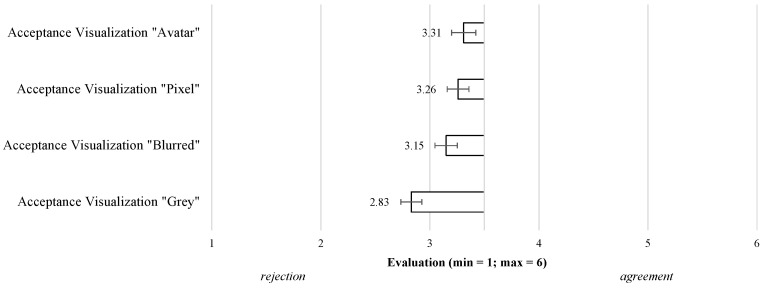
Acceptance of the different visualizations of video-based AAL technology (n = 161).

Subsequent to the video presentation of each visualization, the participants evaluated each visualization mode. Starting with the **Acceptance of the Visualizations** (Figure 7), the results showed that—in line with the overall acceptance (see Section 3.1)—none of the visualization modes was accepted, as all evaluations were lower than the mean of the scale (M = 3.5). In a direct comparison ($F\left(2.39,160\right)=5.49;p<0.01;\eta^{2}=0.03$), the acceptance and intention to use the “Avatar” (M = 3.31; SD = 1.40), “Pixel” (M = 3.26; SD = 1.27), and “Blurred” (M = 3.15; SD = 1.30) visualization modes were only slightly rejected. The “Grey” mode (M = 2.83; SD = 1.22) received the lowest evaluation, indicating a more distinct rejection of this visualization mode.

#### 3.2.3. Affective Evaluations of the Visualization Modes (RQ4)

**Figure 8:**
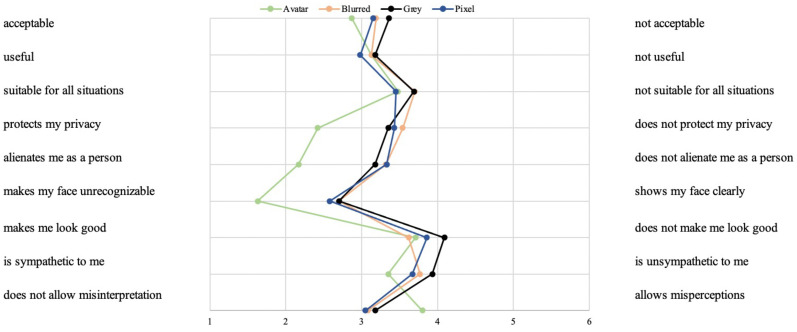
Affective evaluation of different visualizations of video-based AAL technology (n = 161).

In addition to the acceptance of the visualization modes, the examination of a more affective assessment in terms of a semantic differential was aimed at enabling more detailed insights into the motives and reasons for the overall evaluation (see Figure 8). Starting with the first pair of adjectives *acceptable (=1) vs. not acceptable (=6)* (F(2.33,160)=4.34;$p<0.05;\eta^{2}=0.03$), the “Avatar” visualization was most accepted (M = 2.87; SD = 1.61), followed by the “Pixel” (M = 3.15; SD = 1.55) and “Blurred” (M = 3.19; SD = 1.62) modes. The “Grey” (M = 3.36; SD = 1.54) visualization was assessed rather neutrally. Referring to the adjective pairs *useful (=1) vs. not useful (=6)* (F(2.56,160)=0.79;p=0.48,n.s.) and *suitable for all situations vs. not suitable for all situations* (F(2.46,160)=1.63;p=0.07,n.s.) the results did not reveal significant differences between the four visualization modes.

In contrast, the results showed significant differences for the three privacy-related pairs of adjectives. Related to *protects my privacy (=1) vs. does not protect my privacy (=6)* ($F\left(2.66,160\right)=36.64;p<0.01;\eta^{2}=0.19$), the “Avatar” mode (M = 2.42; SD = 1.62) clearly received privacy-protecting evaluations. The “Grey” (M = 3.35; SD = 1.62), “Pixel” (M = 3.43; SD = 1.59), and “Blurred” (M = 3.54; SD = 1.61) visualization modes were evaluated rather neutrally, circulating around the middle of the scale. Further, the results identified a similar pattern for *alienates me as a person (=1) vs. does not alienate me as a person (=6)* (F(2.84,160)=33.31;$p<0.01;\eta^{2}=0.17$). Again, the “Avatar” mode (M = 2.17; SD = 1.61) received more positive evaluations compared to the “Grey” (M = 3.18; SD = 1.61), “Pixel” (M = 3.33; SD = 1.49), and “Blurred” (M = 3.32; SD = 1.54) visualization modes. Referring to *makes my face unrecognizable (=1) vs. shows my face clearly* (F(2.79,160)=32.87;$p<0.01;\eta^{2}=0.17$), the same evaluation pattern was found as well: the “Avatar” mode (M = 1.63; SD = 1.13) showed the best privacy-preserving evaluations compared to clearly less positive evaluations of the “Grey” (M = 2.70; SD = 1.56), “Pixel” (M = 2.58; SD = 1.43), and “Blurred” (M = 2.70; SD = 1.45) visualization modes.

For three other adjectives pairs, the results also identified significant differences between the visualization modes, but to a lower extent. Starting with *makes me look good (=1) vs. does not make me look good (=6)* ($F\left(2.60,160\right)=6.32;p<0.01;\eta^{2}=0.04$), the “Avatar” (M = 3.71; SD = 1.53) and “Blurred” (M = 3.62; SD = 1.30) modes were evaluated rather neutrally. The “Pixel” (M = 3.86; SD = 1.17) and “Grey” (M = 4.09; SD = 1.30) modes tended slightly more toward the negative pole. Related to *is sympathetic to me (=1) vs. is unsympathetic to me (=6)* ($F\left(2.36,160\right)=5.27;p<0.05;\eta^{2}=0.03$), the “Avatar” mode (M = 3.35; SD = 1.69) received a comparably more positive evaluation, followed by the “Pixel” (M = 3.67; SD = 1.56), “Blurred” (M = 3.77; SD = 1.60), and “Grey” (M = 3.93; SD = 1.53) visualization modes. Finally, the results showed significant differences for the adjective pair *does not allow misinterpretation (=1) vs. allows misperceptions (=6)* (F(2.68,160)=14.30;$p<0.01;\eta^{2}=0.08$): here, the “Avatar” visualization (M = 3.80; SD = 1.67) was rated to be more prone to misperceptions compared to the “Grey” (M = 3.18; SD = 1.60), “Pixel” (M = 3.05; SD = 1.45), and “Blurred” (M = 3.08; SD = 1.43) visualization modes.

### 3.3. Does User Diversity Make a Difference? (RQ5)

To analyze whether and to what extent individual characteristics of future users impact the evaluation and acceptance of video-based AAL technologies, a two-step cluster analysis was conducted. It was assumed that groups of future users exist that differ in their evaluation patterns as well as in their individual characteristics. According to the hierarchical cluster analysis based on the three constructs: (1) overall acceptance of video-based AAL technologies, (2) the perceived benefits and (3) the perceived barriers, two clusters were identified as the optimal cluster solution in the data set. In addition to specific differences in the evaluation, both clusters were also characterized by differences in the individual factors, which are introduced first (Table 1).

The identified clusters did not differ regarding demographic characteristics, e.g., age, gender, or educational level. In addition, both clusters did not differ regarding health (e.g., presence of a chronic disease or an impairment/restriction) or living situation. Instead, both clusters differed regarding attitudinal characteristics. Cluster 1 was characterized by a significantly lower perception of privacy needs, whereas Cluster 2 showed higher needs regarding protecting and preserving privacy. Beyond that, technology commitment was significantly higher in Cluster 1 compared to Cluster 2.

**Figure 9:**
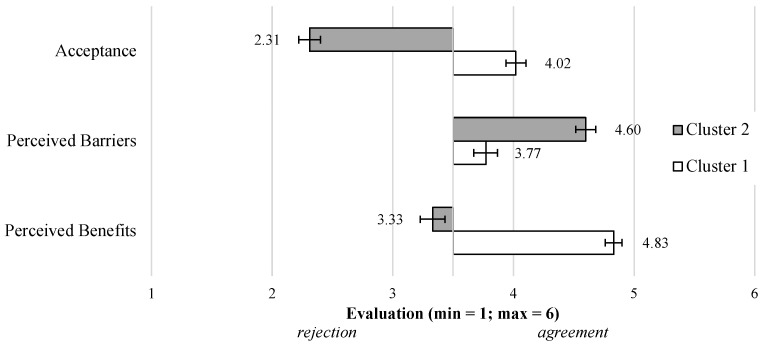
Cluster-specific acceptance of video-based AAL technology (n = 161).

Looking at the basic variables for the cluster segmentation (Figure 9), the three constructs differed fundamentally for both clusters. Starting with the *Acceptance* in terms of an intention to use video-based AAL technology ($F\left(1,160\right)=194.64;p<0.01;\eta^{2}=0.55$), Cluster 1 (M = 4.02; SD = 0.75) showed a positive evaluation, while Cluster 2 (M = 2.31; SD = 0.80) expressed a rejection of using video-based AAL technology. The *Perceived Benefits* ($F\left(1,160\right)=145.90;p<0.01;\eta^{2}=0.48$) were clearly evaluated positively by Cluster 1 (M = 4.83; SD = 0.62), while they were slightly rejected by Cluster 2 (M = 3.33; SD = 0.93). Finally, the *Perceived Barriers* ($F\left(1,160\right)=40.94;p<0.01;\eta^{2}=0.20$) were only slightly confirmed by Cluster 1 (M = 3.77; SD = 0.89), while Cluster 2 (M = 4.60; SD = 0.74) showed a strong agreement with the barriers.

**Figure 10:**
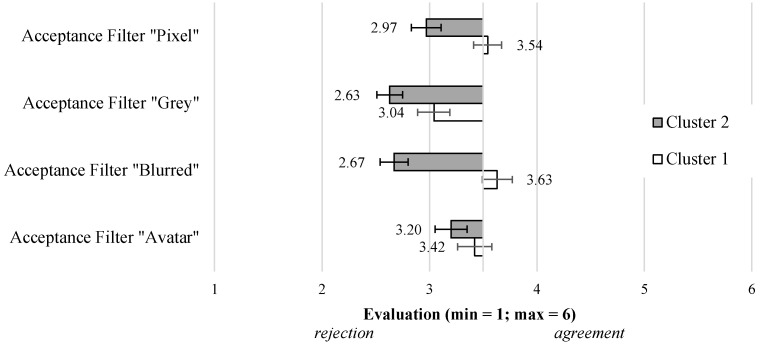
Acceptance of visualization modes depending on the identified clusters (n = 161).

Beyond the cluster-based evaluation differences with regard to the general acceptance of video-based AAL technologies, we analyzed whether the identified clusters also differed in the evaluation of the specific visualization modes. Figure 10 shows that both identified clusters also differed regarding the acceptance of the visualization modes indicating that Cluster 1—with a more positive evaluation and higher acceptance of video-based AAL technologies in general—evaluated the visualization modes more positively or at least less negatively compared to Cluster 2. In more detail, both clusters showed a slight rejection of the “Avatar” mode (F(1,160)=0.95;p=0.33,n.s.), and descriptively, Cluster 2 (M = 3.20; SD = 1.36) was slightly more negative than Cluster 1 (M = 3.42; SD = 1.44). Considering the “Grey” visualization mode ($F\left(1,160\right)=4.57;p<0.01;\eta^{2}=0.03$), the same evaluation pattern occurred, but even stronger: here, Cluster 2 (M = 2.63; SD = 1.06) showed a more negative evaluation and lower acceptance than Cluster 1 (M = 3.04; SD = 1.04). In line with this, the “Pixel” visualization mode ($F\left(1,160\right)=8.52;p<0.01;\eta^{2}=0.05$) was also evaluated more negatively by Cluster 2 (M = 2.97; SD = 1.28) compared to Cluster 1 (M = 3.54; SD = 1.21). Finally, the “Blurred” mode received the most diverse evaluations ($F\left(1,160\right)=24.99;p<0.01;\eta^{2}=0.14$): here, Cluster 1 (M = 3.63; SD = 1.23) showed a slightly positive assessment, while Cluster 2 (M = 2.67; SD = 1.20) expressed a rejection of using this visualization in the context of video-based AAL technologies.

## 4. Discussion

In the following section, the key insights of the study are discussed, focusing on their relevance within the research field. Beyond that, recommendations and implications for the future development and communication of video-based AAL technologies are derived. Finally, the limitations of this study and ideas for future research are elaborated on.

### 4.1. Key Insights, Their Relevance, and Derived Implications

The first research question (RQ1) of this study referred to the general perception and acceptance of video-based AAL technology. In line with previous research, the results of this study confirmed that video-based AAL technologies are evaluated on a neutral, slightly rejecting level, indicating that visual approaches are perceived to be more critical than audio or rather binary technologies [13,14], such as room presence sensors, heat sensors, or humidity sensors [65].

In more detail, the participants acknowledged the potential benefits of using video-based AAL technologies, especially regarding emergency-related aspects, such as notifications in case of emergencies. It is therefore recommended that future information strategies regarding video-based AAL technologies should focus on communicating these highly relevant perceived benefits: thereby, emergency-related functions should be taken as an example and should be explained in a technically correct but transparent, comprehensible, and clear manner.

In addition to the perceived benefits, the results of this study revealed high confirming evaluations of perceived barriers as well, in particular regarding privacy-related aspects, whereas the economic and technical concerns were comparably low. These high confirmations of the (privacy-related) perceived barriers are characteristic of video-based AAL technologies, as previous research clearly identified lower evaluations of these barriers and concerns related to AAL technologies in general [14] or in specific, such as sensor-based AAL systems [65]. The high relevance of privacy-related concerns already suggests that communication and information strategies should focus on privacy handling and data security when video-based AAL technologies are implemented. Here, the technical conditions should be transparently and comprehensively communicated, e.g., in terms of possibilities to turn the system on and off or to grant consent for data access to specific people (e.g., family members, doctors, caregivers). The subsequent discussion of the other research questions will detail the recommendations regarding privacy handling and privacy preservation. To enhance technology uptake, the communication strategies of the single visualization modes should be embedded as part of a holistic design strategy of data visualization of all data captured through the monitoring process [66]. This includes, for instance, effective and efficient data abstractions and layouts, or the addition of feedback loops when interacting with the system.

#### 4.1.1. Influence of Application Contexts

Moving to the second research question (RQ2), it was aimed at an investigation of future users’ preferences when the specific visualization modes were used for different situations in their everyday life. In the present study, three exemplary situations of daily living in older age were prefixed, covering a broad range of situations of life in older age (and being relevant from a technical development perspective). In the first step, the results confirmed that the three situations (“Falling”, “Eating”, and “Undressing”) were clearly perceived differently in terms of the danger attributed to them—intimacy and privacy. These findings highlight that privacy perceptions are strongly context-bounded [43,45,46], with activities possessing an intimate and sensitive character being more privacy-critical especially when it comes to video-monitoring [53]. While these situations represented useful examples to obtain first insights into the users’ preferences in the selection of specific visualization modes, the three situations were not selected and investigated systematically. Indeed, there are many more situations that should be analyzed in detail (e.g., care-related activities, social activities, other intimate activities etc.) [67]. Beyond that, combinations of different situations or activities that are characteristic of everyday life should also be examined. Especially in older age, several safety-critical events may happen during such activity/incident combinations. For instance, a fall can happen during any activity of daily life or a person can eat or drink something that is potentially dangerous for their current health status. These situations should be considered in future investigations.

Nevertheless, the three selected situations first enabled concrete selections of the preferred visualization modes for video-based AAL technologies and resulted in different preference patterns. For the situation of “Eating”, the “Avatar” mode clearly represented the most preferred visualization type: presumably due to the highest level of privacy preservation enabled by this visualization, as it does not allow any conclusions about the colors, textures, and shapes of the monitored person. Further, a third of the participants did not select a visualization mode being of the opinion that this activity should either not be filmed by a camera or they would not permit the original video. These two opposite answers highlight the discrepancy in the trade-offs between protecting the privacy and disclosure of privacy in order to enable functions improving security, autonomy, or independence. For the situation of “Undressing”, the majority of the participants did not want this situation to be filmed by a camera due to the high intimacy and high need for privacy in this situation. However, the “Avatar” mode still represented the most preferred visualization mode. In particular, in this—very intimately perceived—situation, a visualization mode is desired that conceals individual characteristics, such as shape, colors, and textures. Considering the situation of “Falling”, the selection patterns changed in that the majority of the participants would permit the original video. Here, the trade-off between preserving privacy and increasing security turned in favor of increasing security, depending on this—very dangerously perceived—situation. Related to the visualization modes, the “Grey” and the “Avatar” modes were preferred. In this regard, it can be assumed that the “Avatar” mode was in part selected as it still provides the most protection of privacy out of the four alternatives. The “Grey” mode was presumably selected due to the fact that more details can be obtained from a video applying this visualization (i.e., gestures and shapes can be identified) increasing the security in terms of a higher probability of correct detection of falls and emergencies. The “popularity” (even with comparably low ratings) of the avatar across all three situations may be explained in terms of bodily privacy [62,63] and de-identification. In this visualization mode, the entire body image is replaced. This means that inferences on, for example, the skin color and texture, clothing or emotional expressions cannot be made, and even the detection of individual gesture patterns may be much more difficult, if not impossible. Reflecting upon the privacy dimensions of Burgoon [41], control over personal information, control over thoughts, emotions and identity is given in a sense that the authority over these aspects is not given away to the AAL system. The other dimensions of social and physical privacy may only play a marginal role when it comes to visualization preferences. Nonetheless, future studies may investigate these filter options for different situations while assessing the relevance of each of these dimensions of privacy. In addition, as mentioned earlier, combinations of different activities may happen within the same situation. As such, “falling” is very likely to happen during “undressing” or even during “eating”. Given the differing evaluation patterns for the visualization modes for “eating”, “undressing” and “falling”, it is important to investigate participants’ trade-offs when these activities happen simultaneously.

Summarizing these situation-dependent results, it is not that easy to derive generally applicable recommendations. However, the results show that situations of daily living are perceived very differently and that there is no single decision profile that applies equally to all future users of video-based AAL technologies. Therefore, video-based AAL technologies and their visualizations should be individually tailored to the users by enabling them to turn the cameras on and off and to select specific visualization modes depending on the respective context (i.e., situation or activity). This way, future users are free to decide whether to be recorded at all in certain situations or in which manner video footage can be recorded and seen. Beyond that, future communication and information strategies should focus on the trade-off between (i) protecting privacy by using visualizations with a high degree of privacy preservation and (ii) increasing security by choosing visualizations that allow more conclusions to be drawn about situations in terms of representing, e.g., shapes and gestures. Here, both directions should be disclosed and explained in a transparent and comprehensible way.

#### 4.1.2. Influence of Technical Characteristics

The investigation of differences in the acceptance of the specific visualization modes represented the content of the next underlying research question (RQ3). In accordance with RQ1 and confirming previous research [13,14] for specific video-based AAL technologies, all visualization modes received evaluations below the mean of the scale and are thus rather (slightly to even more clearly) rejected than accepted. Beyond the insights of previous work, our results revealed that the “Avatar” mode was most accepted, receiving only slightly rejecting values, whereas the “Grey” visualization mode was the overall least accepted as indicated by even stronger rejections.

The results of the fourth research question (RQ4), which focused on potential differences in the evaluation of the visualization modes, provide explanations for the evaluation differences regarding the acceptance (RQ3). Within the semantic differential, the evaluations showed large differences, in particular regarding privacy-related characteristics for which the “Avatar” mode achieved clearly higher ratings compared to all other modes. Hence, the high potential of privacy preservation of this mode led to its higher acceptance. However, the potential for allowing wrong perceptions was rated highest for the “Avatar” mode as well due to the fact that the concealed gestures, colors, textures, and shapes provide the least information compared to all other visualization modes. Again, these evaluations highlight the complexity of the trade-off between protecting privacy and enhancing security. In contrast to the “Avatar” mode, the “Grey” mode was characterized by the most negative evaluations referring to its acceptance but also to its appeal and sympathy. In terms of communication and information strategies, the characteristics of all visualization modes—their potential, their advantages, but also their drawbacks—should be focused on; this way, future users can be enabled to evaluate the trade-off between privacy and security and to make informed decisions about using or not using such video-based AAL technologies.

#### 4.1.3. Influence of User Characteristics: User Diversity Makes a Difference

In the final step of our study (RQ5), we investigated if there were user groups that differed in their evaluations of video-based AAL technologies. Thereby, we identified two clusters that differed regarding their general perception of video-based AAL technologies. One cluster of participants was characterized as adopters because this group revealed having a positive intention to use video-based AAL technologies, a positive perception of the potential benefits of using the technology, and a rather neutral perception of potential barriers. In contrast, the participants of the second cluster were characterized as rejecters of video-based AAL technology, as they expressed a clearly negative intention to use the technology, a rather neutral evaluation of potential benefits, but a clear confirmation of the potential barriers to using video-based AAL technology. Summarizing these relationships, the adopters were motivated to use video-based AAL technology predominantly driven by the advantages the technology brings along, whereas the rejecters denied the usage of video-based technology based on a high perception of the technology’s potential disadvantages. From a privacy-perception perspective, it seems that participants manage their privacy regulations based on how much weight they put on the benefits and barriers. Seemingly, AAL-rejecters consider barriers, including concerns regarding privacy, as a disturbance in their manner of “*how*” they seek privacy—their privacy regulation management—not enabling them to reach their desired level of privacy. These cluster differences were also visible regarding the evaluation of the specific visualization modes, in which the rejecters clearly showed more negative assessments regarding the acceptance of the specific modes compared to the adopters. However, the adopters’ evaluations also did not result in positive ratings of the visualization modes’ acceptance. This shows that even for people with a positive attitude towards video-based AAL technology, the four visualization modes did not represent an optimal solution for applying video-based AAL technologies. Future research should, therefore, integrate future users right from the beginning to consider their opinions and ideas in the development of other, specifically tailored visualization modes focusing maybe not only on the way video information is presented but also on what exactly it is that is presented (e.g., visualizations for specific parts of the body), following a skin segmentation approach [67].

In addition to the differences in the evaluation patterns, the two clusters were characterized by individual differences as well. The adopters showed a more positive technology commitment, while the rejecters were characterized by a higher privacy perception in terms of higher need for privacy. Apparently, for the adopters, the benefits were quite sufficient to satisfy needs such as autonomy, confiding or contemplation [46], whereas rejecters clearly did not perceive that these privacy needs were met with the benefits at hand. Previous research has already identified that such individual attitudes impact the acceptance of AAL technologies (e.g., [11,68,69]). For video-based AAL technologies, these insights represent new findings, confirming the identified tendencies in previous research. Future information and communication strategies should be aware of the diversity of potential user groups, considering their respective needs and requirements. To reach the more tech-savvy adopting user group described here, it is recommended to highlight the advantages of applying the visualization modes based on the technical functions and requirements. To fulfill the needs of the privacy-aware, rejecting user group, it is of utmost importance to inform about ways of data handling, data access, and privacy protection. This could be achieved by explaining the options of turning on and off the cameras, in general, as well as selecting the visualization modes and providing access to the recorded data for specifically defined persons.

### 4.2. Conclusions, Limitations, and Future Work

This study provided detailed insights into a scenario-based but realistic evaluation of different visualization modes being applied for using video-based AAL technologies. In addition to the general insights into the comparably low acceptance and perception of video-based AAL technology, the visualization modes were specifically evaluated in terms of their acceptance and their potential for privacy preservation, as well as other technical and context-dependent characteristics. Here, the “Avatar” mode was revealed to be the most preferred one enabling the highest (perceived) level of privacy preservation.

In addition to detailed new findings on the acceptance of video-based AAL technology, some limitations have to be considered for future research regarding methodological and sample-related aspects. Starting with a methodological issue, it has to be noted that the survey was comparably long and complex. In particular, watching all video sequences (i.e., original video and four visualization modes) for the three selected activities required some patience from the respondents; the time taken could serve as an explanation for the comparably high rate of incomplete data sets (n = 99). However, this procedure enabled an assessment as realistic as possible, avoiding sequence effects through the randomized design. Considering this study’s sample, its distribution regarding age and gender was quite balanced, whereas the educational level was high. This distribution may be due to the online format of the survey. In addition, the acquisition primarily addressed people who are interested in or committed to the topic of the study and corresponding sub-topics. As a result, the sample could also have an increased proportion of more highly educated people. Here, future studies should try to reach and address all levels of education adequately, e.g., by additionally using paper and pencil surveys. Discussing the age distribution in our sample, we realized our aim of reaching a broad sample covering the whole range of adulthood. The median age (49) showed that we reached older users as well as younger users (representing potential future users as well as a control group). It would be worth specifically examining the group of oldest and frailest users in more detail, i.e., people with disabilities/chronic illnesses, in order to elaborate on their specific requirements and wishes, as this group might benefit from the video assistance the most.

The last aspect refers to the fact that the participants in this study evaluated the usage of video-based AAL technology from their own perspective, imagining the usage within their own home environment. Future studies should investigate other scenarios in order to compare evaluation and acceptance patterns, e.g., perspectives of caring relatives, perspectives of professional caregivers, or the perspective of being an older person in need of care.

## Figures and Tables

**Table 1:** Individual characteristics of both identified clusters.

	Variable	Cluster 1 (n = 81)	Cluster 2 (n = 80)	Statistical Information
Demographics	Age (M, SD)	47.65 (19.18)	46.08 (19.73)	*F*(1, 156) = 0.26 *p* = 0.61, n.s.
	Gender	38.3% male 65.5% female	37.5% male 62.5% female	*F*(1, 160) = 0.003 *p* = 0.95, n.s.
Health	Chronic Disease	27.2% yes 72.8% no	27.5% yes 72.5% no	*F*(1, 160) = 0.002 *p* = 0.96, n.s.
	Impairment/ restriction	13.6% yes 86.4% no	13.8% 86.2%	*F*(1, 160) = 0.001 *p* = 0.98, n.s.
***Attitudinal*** ***Characteristics*** ***(M, SD)***	***Privacy*** ***Perception***	***4.13 (0.90)***	***4.57 (0.96)***	***F(1, 160) = 8.58*** ***p < 0.01*;** $\eta^{2}=0.05$
	***Technology*** ***Commitment***	***4.24 (0.96)***	***3.92 (1.01)***	***F(1, 160) = 4.23*** ***p < 0.05*;** $\eta^{2}=0.03$

## Data Availability

The data that support the findings of this study are available on request from the corresponding author, [J.O.].
